# Impact of a 3-Month Recall Using High-Fidelity Simulation or Screen-Based Simulation on Learning Retention During Neonatal Resuscitation Training for Residents in Anesthesia and Intensive Care: Randomized Controlled Trial

**DOI:** 10.2196/57057

**Published:** 2025-03-21

**Authors:** Anne-Claire Louvel, Cécile Dopff, Gauthier Loron, Daphne Michelet

**Affiliations:** 1 Department of Anesthesia and Intensive Care, Hôpital Bichat-Claude-Bernard, Paris, France; 2 Department of Anesthesia and Intensive Care, Centre Hospitalier Universitaire de Reims, Reims, France; 3 CReSTIC EA 3804, Université de Reims Champagne-Ardenne, Reims, 51100, France; 4 Department of Anesthesia and Intensive Care, Centre Hospitalier Universitaire de Reims, 45 rue Cognacq-Jay, Reims, 51100, France, 33 617861084; 5 C2S laboratory, Université de Reims Champagne-Ardenne, Reims, France

**Keywords:** screen-based simulation, high-fidelity simulation, neonatal resuscitation, pediatric, infant, neonatal, newborns, emergency, urgent, simulation, resuscitation, intensive care, medical education, anesthesia, anesthesiology, high fidelity, educational, student, resident, knowledge retention, learner, teaching, intensive care unit, ICU

## Abstract

**Background:**

Retention capacities are dependent on the learning context. The optimal interval between two learning sessions to maintain a learner’s knowledge is often a subject of discussion, along with the methodology being used. Screen-based simulation could represent an easy alternative for retraining in neonatal resuscitation.

**Objective:**

The aim of the study was to evaluate the benefits of a 3-month recall session using high-fidelity simulation or screen-based simulation, assessed 6 months after an initial neonatal resuscitation training session among anesthesia and intensive care residents.

**Methods:**

All participating anesthesia and intensive care residents were volunteers, and they underwent training in the same session, which included a theoretical course and high-fidelity simulation. The attendees were then randomized into three groups: one with no 3-month recall, one with a high-fidelity simulation recall, and one with a screen-based simulation recall. To reassess the skills of each participant, a high-fidelity simulation was performed at 6 months. The primary outcomes included expert assessment of technical skills using the Neonatal Resuscitation Performance Evaluation score and nontechnical skills assessed by the Anesthesia Non-Technical Skills score. Secondary outcomes included a knowledge quiz and self-assessment of confidence. We compared the results between groups and analyzed intragroup progressions.

**Results:**

Twenty-eight participants were included in the study. No significant differences were observed between groups at the 6-month evaluation. However, we observed a significant improvement in theoretical knowledge and self-confidence among students over time. Regarding nontechnical skills, as evaluated by the Anesthesia Non-Technical Skills score, there was significant improvement between the initial training and the 6-month session in both recall groups (16 vs 12.8, *P*=.01 in the high-fidelity group; 16 vs 13.9, *P*=.05 in the simulation group; 14.7 vs 15.1, *P*=.50 in the control group). For technical skills assessed by the Neonatal Resuscitation Performance Evaluation score, a nonsignificant trend toward improvement was observed in the two recall groups, while a regression was observed in the control group (all *P*s>.05). The increase in students’ self-confidence was significant across all groups but remained higher in the two 3-month recall groups.

**Conclusions:**

Initial neonatal resuscitation training for anesthesia and intensive care residents leads to improved knowledge and self-confidence that persist at 6 months. A 3-month recall session, whether through high-fidelity simulation or screen-based simulation, improves nontechnical skills (eg, situation management and team communication) and technical skills. Screen-based simulation, which saves time and resources, appears to be an effective educational method for recall after initial training. The study outcomes justify the need for further studies with larger sample sizes to confirm the promising role of serious games in educational programs for medical students.

## Introduction

Neonatal resuscitation is a potentially critical situation that requires training. Nearly 10% of newborns and 80% of infants weighing less than 1500 g require resuscitation at birth, and the quality of care provided during the first minute of life is directly related to the prognosis [1-3]. Theoretical knowledge based on current recommendations and practical training are key for ensuring optimal neonatal resuscitation. Anesthesia and intensive care physicians may need to intervene when a pediatrician is not immediately available, for instance, to assist with intubation. Therefore, we decided to train and prepare our residents for such scenarios.

Screen-based simulation is an emerging simulation-based training tool for health care professionals, ensuring comparable learning effectiveness to traditional learning methods [4]. Recent developments in computer science have allowed the creation of highly realistic medical digital simulators, improving the acquisition of both knowledge and nontechnical skills, as well as technical skills [5,6]. A screen-based simulator (NRP eSim), designed by Laerdal Medical in collaboration with the American Academy of Pediatrics is included in the Neonatal Resuscitation Program as one of the six educational components of the 7th edition of the Neonatal Resuscitation Program training program [7]. In the neonatal resuscitation context, technical skills refer to diagnostic and therapeutic actions, whereas nontechnical skills include leadership, teamwork, communication, and task management.

Few studies have already assessed knowledge retention following screen-based simulation training. While results are relatively positive when evaluated within one or two months after simulation [8,9], they suggest lower effectiveness compared to traditional learning methods when assessed after 6 months [10].

The question of knowledge retention is still debated, and the ideal interval for refresher training remains uncertain, regardless of the method [11]. Recent studies indicate the need for regular training for complex procedures, such as the management of cardiac arrest with intervals of 3 to 6 months [12], or even monthly [13].

The objective of our study is to analyze the retention of knowledge and skills at 6 months after an initial training in neonatal resuscitation for anesthesia and intensive care residents, with or without a 3-month recall training session using either screen-based simulation or high-fidelity (HF) simulation.

## Methods

### Ethical Considerations

This randomized controlled simulation study was conducted from February 2021 to November 2021 at the University Hospital of Reims, France. The study was approved by the CERAR (Ethics Committee for Research in Anesthesia Resuscitation; approval number: 00010254-2021-048). All students provided written informed consent (Multimedia Appendix 1). All data were anonymized using research numbers. No compensation was provided to the residents who volunteered to participate in the study. Consent was provided for the video recording and analysis of primary and secondary outcomes. The CONSORT-eHealth checklist is provided (Checklist 1) [14].

### Participants

Volunteer participants were recruited from third-, fourth-, and fifth-year anesthesiology and intensive care residency classes in Reims. None had previous courses or training in neonatal resuscitation. They were informed about the study via email from the teaching supervisor (DM). To confirm their participation, they had to ensure that they could be present at all times during the study (ie, 3 and 6 months).

### Design of the Study

**Figure 1.**
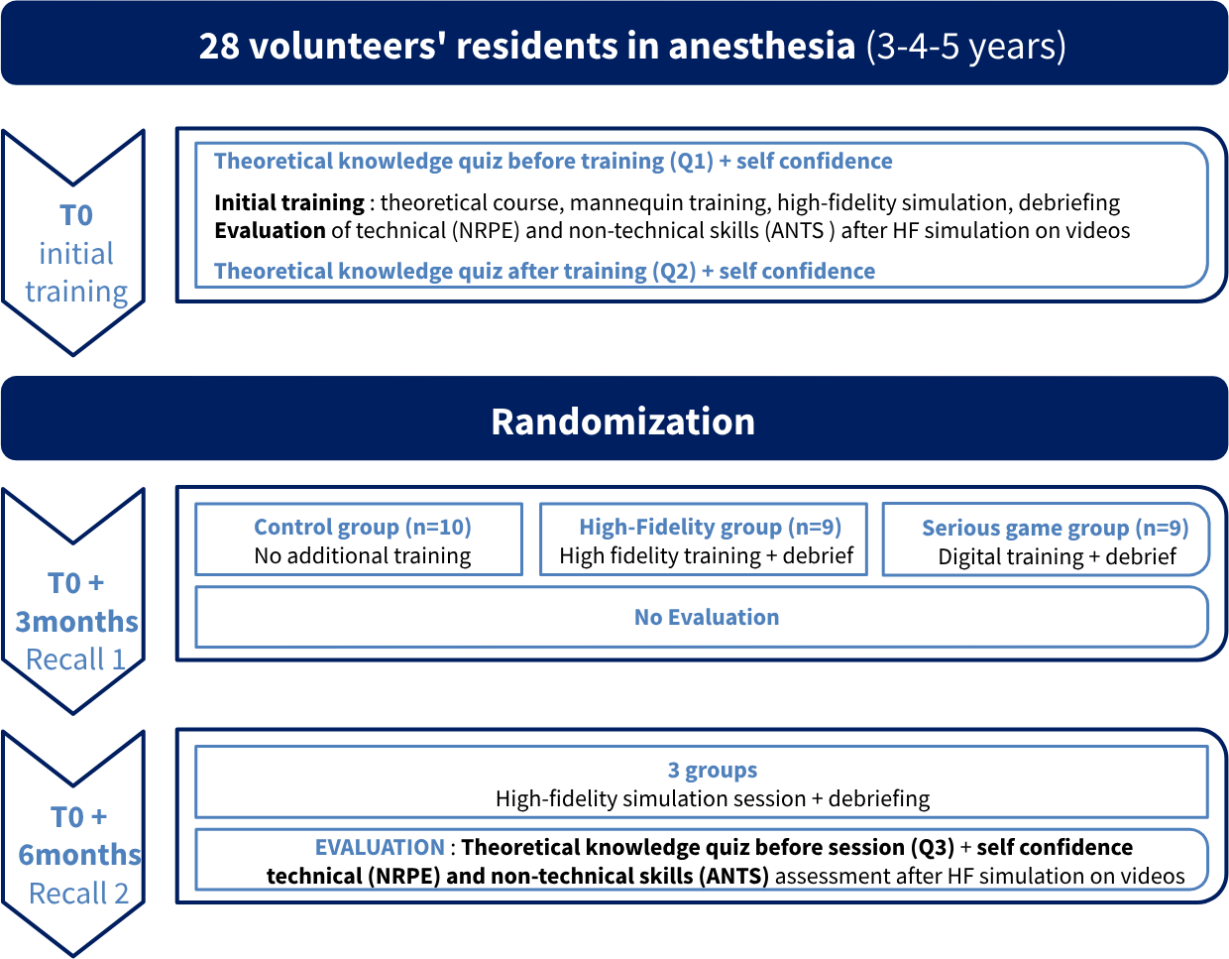
Experimental design. ANTS: Anesthesia Non-Technical Skills; HF: high-fidelity; NRPE: Neonatal Resuscitation Performance Evaluation; T0: time zero.

The experimental design is summarized in Figure 1.

### Initial Training

The initial training was conducted by a pediatric intensivist and an anesthetist. It consisted of a 45-minute course on the basics and essential knowledge required for neonatal resuscitation based on the International Liaison Committee on Resuscitation (ILCOR) 2020 recommendations [15]. Subsequently, the interns practiced ventilation using a low-fidelity mannequin using the Neopuff (Fisher & Paykel Healthcare), intubation, and cardiac massage. Subsequently, they individually participated in an HF simulation that included a presentation of the material, briefing, scenario execution, and debriefing (see scenario details in Multimedia Appendix 2). The simulation was conducted in a real-life delivery room setting at Reims University Hospital using the SimNewBorn mannequin (Laerdal Medical). The simulation sessions were recorded for later analysis.

Residents were randomized into three parallel groups (randomization.com) in a 1:1:1 ratio. The control group did not receive a recall session at 3 months. The two intervention groups received a recall session at 3 months after the initial session using either serious game (SG) screen-based simulation or HF simulation.

### Three-Month Recall

#### HF Simulation

Each participant individually participated in an HF simulation using the SimNewBorn mannequin in the delivery room. The scenario is provided in Multimedia Appendix 2. The session lasted approximately 45 minutes, including briefing, scenario execution, and debriefing with one of the study trainers. The material used was identical to that of the initial training, and participants were given a 5-minute refresher before the simulation.

#### Screen-Based Simulation

**Figure 2.**
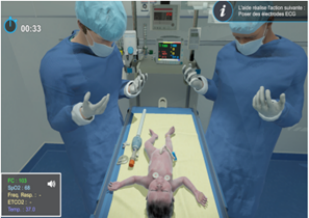
Screenshot of interface and virtual environment of the PerinatSims screen-based simulation.

Each participant performed an individual screen-based simulation consisting of a 10-minute tutorial explaining the various possible actions and a scenario. The screen-based simulation, PerinatSims, was designed by Medusims. It features a 3D virtual environment of a delivery room, with a newborn placed on a neonatal resuscitation table (Figure 2). The simulation used a point-and-click interface with a first-person point of view.

The scenario was similar to that used for the HF group (see Multimedia Appendix 2) and was followed by a digital debriefing. The session lasted 45 minutes and was conducted in the presence of a trainer to prevent technical issues from affecting the research. Participants logged onto the digital simulator on a computer at the hospital, with access granted for the duration of the study.

### Six-Month Evaluation

#### Overview

The final evaluation for each participant of the study involved an HF simulation, initiated with a briefing and presentation of the material, followed by scenario execution using the SimNewBaby Mannequin, and debriefing. The evaluation scenario is provided in Multimedia Appendix 2. Simulation sessions were recorded for later analysis.

The potential exposure of each participant to real-life neonatal resuscitation cases or additional training during the 6-month study period was controlled and monitored.

#### Primary End Points: Comparison of Nontechnical and Technical Skills Retention

Two independent, blinded anesthetist raters retrospectively evaluated the technical and nontechnical skills by analyzing video recordings from the initial and final sessions.

Technical skills were assessed by the Neonatal Resuscitation Performance Evaluation (NRPE) scoring system (20 points) adapted for each scenario [16], and nontechnical skills using the Anesthesia Non-Technical Skills (ANTS) observation system, which includes four categories (ie, situation awareness, task management, team work, and decision-making) [17]. ANTS is a validated tool used to assess nontechnical skills in various simulation situations, ranging from emergency training for medical students [18] to neonatal resuscitation for midwives using a specific modified ANTS version [19]. The ANTS scores were recorded as the overall category scores on a scale of 1‐4 (poor performance: 1 to good performance: 4). The global score (out of 16 points) is presented as a 20-point scale in the Results section, as described by the authors of the ANTS. Interrater reliability calculations were performed.

#### Secondary End Points

##### Comparison of Knowledge Retention

Knowledge was assessed by a validated questionnaire containing 25 single or multiple-choice questions based on ILCOR recommendations [15]. Assessments were performed at Q1: beginning of the initial training; Q2: end of the initial training; and Q3: beginning of the 6-month evaluation.

##### Comparison of the Self-Confidence Auto-Evaluation

The self-confidence question assessed residents’ perception of their performance, based on the question, “How much are you confident in your capability to organize and execute a neonatal resuscitation?” The responses were recorded using a 6-point Likert scale, ranging from “not at all confident” (scored as 0) to “very confident” (scored as 5) [20]. Self-confidence was assessed at the end of the initial training and the 6-month evaluation session.

### Data Collection

Data were collected directly on paper for the knowledge and self-confidence questionnaires, as well as for clinical and demographic information. The primary outcome measures were assessed via expert evaluation of the video recordings a posteriori.

### Statistical Analysis

Based on a previous study, a baseline ANTS score of approximately 12/20 was expected [21], with a target improvement to 16/20 through recall interventions. With an alpha risk of 5% and 80% power, the number of participants required per group was 9.

The statistical hypothesis tested whether SG and HF simulation were equivalent in reactivating skills and knowledge, with both differing from the control group. Data are presented as median (Q1-Q3) for continuous data, given the small sample size. Interrater reliability calculations were performed for both evaluations, showing good agreement between the two raters (kw>0.66; *P*=.01) [22]. The statistical analyses were conducted using the Friedman test and Wilcoxon test for paired samples, and the Kruskal-Wallis test for intergroup comparisons. All tests were two-tailed, with statistical significance considered at *P*<.05. Statistical analyses were performed using SPSS software (version 25.0; IBM Corp).

## Results

### Participant Characteristics

**Figure 3.**
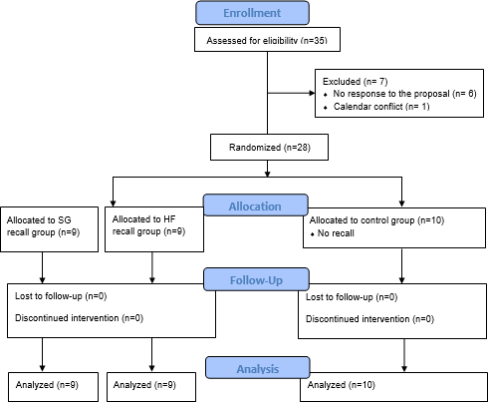
CONSORT flowchart of participant enrollment, allocation, follow up, and analysis. CONSORT: Consolidated Standards of Reporting Trials; HF: high-fidelity; SG: serious games.

Twenty-eight participants were included in the study; 10 were randomly assigned to the control group, 9 to the HF simulation 6-month recall group (HF), and 9 to the screen-based simulation 6-month recall group (SG). All were anesthesiology and intensive care residents in their third, fourth, or fifth year of a 5-year training program in France. Seventeen participants (60%) were men, with a median age of 28 years (IQR 27-31). Baseline demographic and clinical characteristics are presented in Table 1. All participants completed the study (CONSORT [Consolidated Standards of Reporting Trials] flow diagram is provided) (Figure 3).

Seventy-five percent (21/28) of the participants had previously participated in training involving simulation. Twenty-one participants were familiar with HF simulation, and 15 had prior experience with a SG, though none had training in neonatal resuscitation. No participants had encountered or participated in a real neonatal resuscitation during the 6-month study period or benefited from other training. The findings are presented in Table 2.

**Table 1.** Baseline demographic and clinical characteristics of each group.

Variables	HF^a^ group (n=9)	SG^b^ group (n=9)	Control group (n=10)
Age (years), median (IQR)	27.5 (27-29)	30 (29-31)	28 (27-32)
Year of anesthesia training (out of 5), median (IQR)	3.5 (3-5)	4.5 (3-5)	4 (3-5)
Sexes, n (%)			
Female	5 (56)	2 (22)	4 (40)
Male	4 (44)	7 (78)	6 (60)
Previous experience in HF^a^ simulation, n (%)	5 (56)	8 (89)	8 (80)
Previous experience in SG^b^ simulation, n (%)	4 (44)	5 (56)	6 (60)

a HF: High-fidelity.

b SG: Serious game screen-based.

**Table 2.** Comparison of the study parameters within groups using the Friedman and Wilcoxon tests for paired samples.

Participants and end points	Preinitial training, median (IQR)	Postinitial training, median (IQR)	6-month evaluation, median (IQR)	*P* value
Overall (N=28)				
Quiz (20 points)	10 (8-11)	16 (14-16)	13.5 (11.25-15)	.001
Confidence (5 points)	0 (0-1)	3 (2-3)	2 (2-3)	<.001
NRPE^a^ (20 points)	—^b^	14.6 (12.6-15.9)	14.4 (12.4-16)	.86
ANTS^c^ (20 points)	—^b^	14.5 (12.8-15.7)	15.8 (14.6-17)	.005
HF^d^ group (n=9)				
Quiz (20 points)	9 (7-11.5)	16 (15-17.5)	12.5 (11-14.75)	.001
Confidence (5 points)	0 (0-1)	3 (2-3.5)	3 (2-3)	.004
NRPE (20 points)	—	13.3 (12.3-15.9)	14.9 (11.9-17.2)	.50
ANTS (20 points)	—	12.8 (11.9-15.6)	16 (14.8-17.7)	.01
SG^e^ group (n=9)				
Quiz (20 points)	9 (8-11)	16 (13.5-17.5)	13 (10.5-15)	.001
Confidence (5 points)	0 (0-1.5)	3 (1.5-3)	2.5 (2-3.75)	.002
NRPE (20 points)	—	14.5 (12.1-15.3)	15 (14.1-15.9)	.33
ANTS (20 points)	—	13.9 (12.8-15.8)	16 (15.4-17.1)	.05
Control group (n=10)				
Quiz (20 points)	10 (8.75-11.25)	15 (14-15)	14 (13.25-14.75)	.001
Confidence (5 points)	0 (0-1)	2.5 (1-3)	2 (1-2.75)	.003
NRPE (20 points)	—	15.7 (14.7-16.5)	12.6 (11.9-13.9)	.21
ANTS (20 points)	—	15.1 (14.7-15.6)	14.7 (13.4-15.6)	.50

a NRPE: Neonatal Resuscitation Performance Evaluation.

b Not applicable.

c ANTS: Anesthesia Non-Technical Skills.

d HF: High-fidelity.

e SG: Serious game screen-based simulation.

### Primary End Points: Comparison of Technical and Nontechnical Skills Retention

#### Nontechnical Skills (ANTS)

Nontechnical skills, assessed by the ANTS score, showed a significant improvement between the initial training and 6-month session in the two groups that received recall training session.

No significant differences were observed between the groups (*P*=.15).

#### Technical Skills (NRPE)

Technical skills assessed by the NRPE score indicated a nonsignificant trend toward improvement in the two recall groups, whereas a decline was observed in the control group.

No significant differences were observed between the groups (*P*=.19).

### Secondary End Points

#### Comparison of Knowledge Retention

Knowledge scores improved across all groups between the beginning of the study and the end of the initial training. At 6 months, knowledge decreased in all groups but remained higher than the initial level.

No significant differences were observed between the groups (*P*=.12).

#### Comparison of Self-Confidence Evaluation

The increase in students’ self-confidence was significant in all groups between the initial training and 6-month recall; the increase was more pronounced in the two groups that received a 3-month recall session.

## Discussion

### Principal Findings

This study highlights the benefits of a recall session 3 months after an initial neonatal resuscitation training for novice anesthesia and intensive care residents. Regardless of the recall technique—whether an SG or HF simulation—learning retention improved at 6 months.

The importance of knowledge and skill maintenance has been demonstrated several times in medical education, although no ideal interval or optimal method has been defined. A significant loss of skills over time is observed in the absence of continuous training [23,24]. Short, systematic, and regularly repeated training sessions help to optimize the retention of skills, as the consolidation of learning is based on repetition [25,26]. Retention is a marker that is regularly used to assess the quality of learning. The literature demonstrates a significant variability in the duration of retention depending on the type of skills taught and the learner population. In an HF simulation study on the cricothyroidotomy technique involving already qualified anesthetists, retention lasted up to a year after training [27]. This may be attributed to the complexity of the taught task and the high expertise of the learners. However, for a group of novices training in new technical skills, retention can be significantly shorter [28]. Therefore, the teaching format, learners, and the type of skill taught all have an impact on the duration of retention.

Screen-based simulation serves as an alternative to HF simulation or an interesting addition to training programs, as it represents an innovative contribution in line with the expectations of newer generations. It offers flexibility in terms of usage and learning methods—whether individual or group-based, remote or in-person—while preserving a safe environment for immersive and realistic training that is conducive to making mistakes. Additionally, screen-based simulation is less expensive than HF simulation [29]. A recent study examined its implementation to facilitate the development of medical teaching programs using screen-based simulation [30].

Few studies have assessed retention of learning after screen-based simulation training in the medical field. In aviation, one study found better knowledge retention after immersive digital simulator training compared to conventional training tools after one week [31]. In a medical study, the contribution of screen-based simulation was evaluated in comparison to traditional teaching for head and neck physiology and anatomy for second-year medical students. Knowledge retention, assessed through a questionnaire at 6 months, was similar between groups; the traditional teaching group showed progression over time, whereas the screen-based simulation group did not [10]. Another study involving nursing students compared knowledge retention after a 45-minute course on managing respiratory distress, followed by either screen-based simulation or low-fidelity simulation. Screen-based simulation resulted in superior knowledge retention at two months [32]. Additionally, a recent study demonstrated the potential of an online SG as an effective learning tool for improving and retaining knowledge related to the diagnosis and treatment planning of oral lesions, with good knowledge retention observed at one week [33].

Our study is the first to analyze the impact of a 3-month recall session on the retention of learning at 6 months and to compare two recall techniques: screen-based simulation and HF simulation. It is important to emphasize that both recall groups benefitted from identical training durations at 3 months (45 min in total), allowing for a direct comparison of the training methods. Our findings confirm the benefits of a 3-month recall session, with improvement in learning in both recall groups, unlike the control group without recall, in which skills declined.

A 3-month recall appears to enable an improvement in knowledge, particularly in technical and nontechnical skills and participants’ self-confidence. The only element that progressed similarly in the control group was the knowledge questionnaire score, though this may be the least reliable criterion, as learners had the opportunity to review the ILCOR algorithm before participating in the 6-month session. Assessing skills is a more discriminating measure of learning retention.

### Limitations of the Study

The main limitation of our study is the sample size. The study involved voluntary third-, fourth-, and fifth-year anesthesia and intensive care residents, resulting in a limited number of eligible participants (28 out of 35), which corresponds to the sample size calculation but did not allow us to detect any differences between the groups. Notably, the baseline level of the ANTS score was higher than expected, particularly in the control group. However, our population was homogeneous, consisting of neonatal resuscitation novices, and we were able to follow them over the 6 months of the study.

Although this study is a good reflection of a typical educational program, our findings remain to be confirmed using larger cohorts. Additionally they should be validated using other pedagogical themes to refine the role of screen-based simulation in educational pathways and to assess the effect of different methods on the long-term retention of learning.

### Conclusion

A 3-month recall after initial neonatal resuscitation training for novice residents results in improved learning retention at 6 months, regardless of the teaching technique used—numerical or HF simulation. While our findings should be confirmed by future studies with larger sample sizes, they support the value and feasibility of incorporating screen-based simulation into training programs.

## Supplementary material

10.2196/57057 Multimedia Appendix 1 Informed written consent (in French).

10.2196/57057 Multimedia Appendix 2 Simulation scenario.

10.2196/57057 Checklist 1 CONSORT-eHEALTH checklist (V 1.6.1).

## Abbreviations

ANTS: Anesthesia Non-Technical Skills
CONSORT: Consolidated Standards of Reporting Trials
HF: high-fidelity
ILCOR: International Liaison Committee on Resuscitation
NRPE: Neonatal Resuscitation Performance Evaluation
SG: serious game

## References

[1] Ryan CA, Clark LM, Malone A, Ahmed S (1999). The effect of a structured neonatal resuscitation program on delivery room practices. Neonatal Netw.

[2] Curran VR, Aziz K, O’Young S, Bessell C (2004). Evaluation of the effect of a computerized training simulator (ANAKIN) on the retention of neonatal resuscitation skills. Teach Learn Med.

[3] Pearlman SA, Zern SC, Blackson T, Ciarlo JA, Mackley AB, Locke RG (2016). Use of neonatal simulation models to assess competency in bag-mask ventilation. J Perinatol.

[4] Gentry SV, Gauthier A, L’Estrade Ehrstrom B (2019). Serious gaming and gamification education in health professions: systematic review. J Med Internet Res.

[5] Barré J, Michelet D, Job A (2019). Does repeated exposure to critical situations in a screen-based simulation improve the self-assessment of non-technical skills in postpartum hemorrhage management?. Simul Gaming.

[6] Aster A, Laupichler MC, Zimmer S, Raupach T (2024). Game design elements of serious games in the education of medical and healthcare professions: a mixed-methods systematic review of underlying theories and teaching effectiveness. Adv Health Sci Educ Theory Pract.

[7] Sawyer T, Umoren RA, Gray MM (2017). Neonatal resuscitation: advances in training and practice. Adv Med Educ Pract.

[8] Kanthan R, Senger JL (2011). The impact of specially designed digital games-based learning in undergraduate pathology and medical education. Arch Pathol Lab Med.

[9] Barré J, Michelet D, Truchot J, Cabon P, Tesniere A (2021). Midwifery students’ retention of learning after screen-based simulation training on neonatal resuscitation: a pilot study. BMJ Simul Technol Enhanc Learn.

[10] Rondon S, Sassi FC, Furquim de Andrade CR (2013). Computer game-based and traditional learning method: a comparison regarding students’ knowledge retention. BMC Med Educ.

[11] Au K, Lam D, Garg N (2019). Improving skills retention after advanced structured resuscitation training: a systematic review of randomized controlled trials. Resuscitation.

[12] Monsieurs KG, Nolan JP, Bossaert LL (2015). European Resuscitation Council Guidelines for Resuscitation 2015: Section 1. Executive summary. Resuscitation.

[13] Anderson R, Sebaldt A, Lin Y, Cheng A (2019). Optimal training frequency for acquisition and retention of high-quality CPR skills: a randomized trial. Resuscitation.

[14] Eysenbach G, CONSORT-EHEALTH Group (2011). CONSORT-EHEALTH: improving and standardizing evaluation reports of Web-based and mobile health interventions. J Med Internet Res.

[15] (2023). Réanimation néonatale en salle de naissance. Réseaux de santé de Champagne-Ardenne.

[16] van der Heide PA, van Toledo-Eppinga L, van der Heide M, van der Lee JH (2006). Assessment of neonatal resuscitation skills: a reliable and valid scoring system. Resuscitation.

[17] Fletcher G, Flin R, McGeorge P, Glavin R, Maran N, Patey R (2003). Anaesthetists’ Non-Technical Skills (ANTS): evaluation of a behavioural marker system. Br J Anaesth.

[18] Hagemann V, Herbstreit F, Kehren C (2017). Does teaching non-technical skills to medical students improve those skills and simulated patient outcome?. Int J Med Educ.

[19] Cavicchiolo ME, Cavallin F, Staffler A (2019). Decision making and situational awareness in neonatal resuscitation in low resource settings. Resuscitation.

[20] Roh YS, Lee WS, Chung HS, Park YM (2013). The effects of simulation-based resuscitation training on nurses’ self-efficacy and satisfaction. Nurse Educ Today.

[21] Michelet D, Barre J, Truchot J, Piot MA, Cabon P, Tesniere A (2020). Effect of computer debriefing on acquisition and retention of learning after screen-based simulation of neonatal resuscitation: randomized controlled trial. JMIR Serious Games.

[22] Randolph JJ (2008). Online kappa calculator [computer software]. Justus Randolph.

[23] Torrelles Nadal C, Paris Mañas G, Sabrià Bernadó B, Alsinet Mora C (2015). Assessing teamwork competence. Psicothema.

[24] Granry JC (2012). État de l’art (National et International) En Matière de Simulation Dans Le Domaine de La Santé Dans Le Cadre Du Développement Professionnel Continu (DPC) et de La Prévention Des Risques Associés Aux Soins.

[25] McGaugh JL (1999). The perseveration-consolidation hypothesis: Mueller and Pilzecker, 1900. Brain Res Bull.

[26] McGaugh JL (2000). Memory--a century of consolidation. Science.

[27] Boet S, Borges BCR, Naik VN (2011). Complex procedural skills are retained for a minimum of 1 yr after a single high-fidelity simulation training session. Br J Anaesth.

[28] Moulton CAE, Dubrowski A, Macrae H, Graham B, Grober E, Reznick R (2006). Teaching surgical skills: what kind of practice makes perfect?: a randomized, controlled trial. Ann Surg.

[29] Haerling KA (2018). Cost-utility analysis of virtual and mannequin-based simulation. Simul Healthc.

[30] Ebm C, Del Pozo C, Barbarello A, Poli G, Brusa S (2024). Unleashing excellence: using a project management approach to effectively implement a simulation curriculum to improve residents’ preparedness. BMC Med Educ.

[31] Chittaro L, Buttussi F (2015). Assessing knowledge retention of an immersive serious game vs. a traditional education method in aviation safety. IEEE Trans Vis Comput Graph.

[32] Padilha JM, Machado PP, Ribeiro A, Ramos J, Costa P (2019). Clinical virtual simulation in nursing education: randomized controlled trial. J Med Internet Res.

[33] Buajeeb W, Chokpipatkun J, Achalanan N, Kriwattanawong N, Sipiyaruk K (2023). The development of an online serious game for oral diagnosis and treatment planning: evaluation of knowledge acquisition and retention. BMC Med Educ.

