# Supplementary material for: Impact of a 3-Month Recall Using High-Fidelity Simulation or Screen-Based Simulation on Learning Retention During Neonatal Resuscitation Training for Residents in Anesthesia and Intensive Care: Randomized Controlled Trial
Source: JMIR Serious Games. 2025 Mar 21;13:e57057. doi: 10.2196/57057 (PMC11952274; doi:10.2196/57057)
Supplement: Multimedia Appendix 1 [file games-v13-e57057-s001.doc]

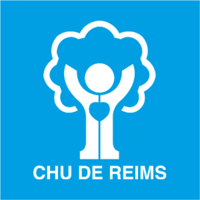
**Consentement de participation à la recherche**

**Autorisation de droit à l'image et/ou de la voix**

**Simulation** **CHU de Reims**

### Objet : Consentement de participation/Autorisation de photographier/filmer et publier des images et/ou voix

Je, soussigné,

**Nom :**…………………………… **Prénom**:……………………………………………

Fonction : …………………………………………………………………………………………

Certifie participer volontairement au projet de recherche « simulation en réanimation néonatale » mené par Gauthier Loron, Cécile Dopff et Daphné Michelet,

Autorise, à titre gratuit l’équipe de simulation du CHU de Reims:

- à me photographier, filmer/et ou m’enregistrer lors de de la réalisation du scénario de simulation ou pendant le débriefing.

- à diffuser la ressource à titre gracieux pour une durée de 10 ans à partir de la date de signature de cette autorisation à l’intérieur et à l’extérieur du CHU de Reims, à l’exclusion de toute utilisation publicitaire ou commerciale et dans le respect du droit moral.

Je m’engage à ne pas tenir responsable la personne ou structure précitée ainsi que ses représentants et toute personne agissant avec sa permission pour ce qui relève de la possibilité d’un changement de cadrage, de couleur et de densité qui pourrait survenir lors de la reproduction.

J’ai lu et compris toutes les implications de cette autorisation.

Fait à ………………………….. Le ………………………….

Signature :
