# Supplementary material for: Impact of a 3-Month Recall Using High-Fidelity Simulation or Screen-Based Simulation on Learning Retention During Neonatal Resuscitation Training for Residents in Anesthesia and Intensive Care: Randomized Controlled Trial
Source: JMIR Serious Games. 2025 Mar 21;13:e57057. doi: 10.2196/57057 (PMC11952274; doi:10.2196/57057)
Supplement: Multimedia Appendix 2 [file games-v13-e57057-s002.docx]

**Appendix**

**Scenario 1 :**

You are an anesthetist on call at a level 1 maternity hospital, with no pediatrician on site. Madam A, 28 years old, 39-weeks pregnant and primiparous, presents at the maternity emergency room for abundant metrorrhagia on the low inserted placenta. An emergency cesarean section (red code) under general anesthesia is quickly decided. The pediatrician has been called but will be able to be there only in 15 minutes. Once general anesthesia is induced, the mother’s condition is stable. She can be monitored by the anesthesist nurse and you are preparing yourself to welcome the child with a midwife who has just been graduated and hired right out of school.

She asks you to take care of the new born because she has no experience.

**Scenario 2 :**

You are an anesthetist on call at a level 1 maternity hospital, with no pediatrician on site. Madam A, 30 years old, 2nd gesture, presents in spontaneous labor at 39 weeks' gestation. The newborn presented with severe alterations to the fetal heart rate at the end of labor. A suction cup was used and meconium amniotic fluid was found. At birth, the newborn, Clémentine, was impregnated with thick greenish meconium amniotic fluid. She weighed approximately 3.5 kg. The pediatrician has been called but will only be able to be on site in 15 minutes. You welcome the child with a midwife who has just been taken on from school. She asks you to take charge of the child as she has no experience.

**Scenario 3 :**

You are an anesthetist on call at a level 1 maternity hospital, without any pediatrician on site. Madam A, aged 30, primiparous at 39 weeks' gestation, is admitted to the emergency caesarean section with a code red for an altered fetal heart rate (bradycardia at 50/min). A large HRP was seen on ultrasound. The pediatrician has been called but will not be able to be on site for another 15 minutes. The mother's condition is stable once the general anesthesia has been induced, she is being monitored by the nurse anaesthetist and you are preparing to welcome the newborn with a midwife who has just been recruited from school. She asks you to take charge of the child as she has no experience.
